# Human Immunodeficiency Virus Type 1 Vif causes dysfunction of Cdk1 and CyclinB1: implications for cell cycle arrest

**DOI:** 10.1186/1743-422X-8-219

**Published:** 2011-05-11

**Authors:** Keiko Sakai, R Anthony Barnitz, Benjamin Chaigne-Delalande, Nicolas Bidère, Michael J Lenardo

**Affiliations:** 1 Laboratory of Immunology, National Institutes of Allergy and Infectious Diseases, National Institutes of Health, Bethesda, Maryland, USA; 2 Division of Viral Immunology, Center for AIDS Research, Kumamoto University, Kumamoto, Japan; 3 Department of Pediatric Oncology, Dana-Farber Cancer Institute, Harvard Medical School, Boston, Massachusetts, USA; 4 Institut National de la Santé et de la Recherche Médicale, Unité 542, Université Paris-Sud, Hôpital Paul Brousse, Villejuif, France

## Abstract

The two major cytopathic factors in human immunodeficiency virus type 1 (HIV-1), the accessory proteins viral infectivity factor (Vif) and viral protein R (Vpr), inhibit cell-cycle progression at the G2 phase of the cell cycle. Although Vpr-induced blockade and the associated T-cell death have been well studied, the molecular mechanism of G2 arrest by Vif remains undefined. To elucidate how Vif induces arrest, we infected synchronized Jurkat T-cells and examined the effect of Vif on the activation of Cdk1 and CyclinB1, the chief cell-cycle factors for the G2 to M phase transition. We found that the characteristic dephosphorylation of an inhibitory phosphate on Cdk1 did not occur in infected cells expressing Vif. In addition, the nuclear translocation of Cdk1 and CyclinB1 was disregulated. Finally, Vif-induced cell cycle arrest was correlated with proviral expression of Vif. Taken together, our results suggest that Vif impairs mitotic entry by interfering with Cdk1-CyclinB1 activation.

## Findings

HIV-1 infection results in cell cycle arrest at the G2 phase accompanied by massive CD4^+ ^T-cell death. Amongst the HIV-1 proteins, Vpr has been a major focus of studies for cytopathicity and G2 cell cycle arrest [1,2]. We recently showed that Vif also causes CD4^+ ^T-cell death and G2 arrest during HIV-1 infection, unveiling a connection between virus-induced cell cycle arrest and cytopathicity [3]. Whereas Vpr-induced G2 blockade has been extensively studied [4-14], how Vif causes cell cycle arrest remains poorly defined [3,15-17]. Here, we studied the effect of Vif expression during HIV-1 infection *in vitro *on important mitotic regulatory proteins.

The activation and nuclear accumulation of the Cdk1-CyclinB1 kinase complex, also known as mitosis promoting factor (MPF), are key molecular events during G2/M-phase transition [18-21]. Cascades of phosphorylation and dephosphorylation govern these events at the late G2 phase. Once cells commit to mitotic entry, the Cdc25C phosphatase activates Cdk1 by removing two inhibitory phosphates from Thr14 and Tyr15 [22-28]. The subsequent assembly of an activated Cdk1-CyclinB1 complex initiates a positive feedback loop by phosphorylating Cdc25C, which increases its enzymatic activity [29]. Nuclear accumulation of MPF requires phosphorylation of CyclinB1 in the cytoplasmic retention sequence (CRS) [30-34], possibly by polo-like kinase 1 (PLK1) [35]. As a result of these events, active MPF accumulates in the nucleus and phosphorylates nuclear lamins, thereby ensuring nuclear envelope disassembly and the initiation of mitosis [36-38].

**Figure 1:**
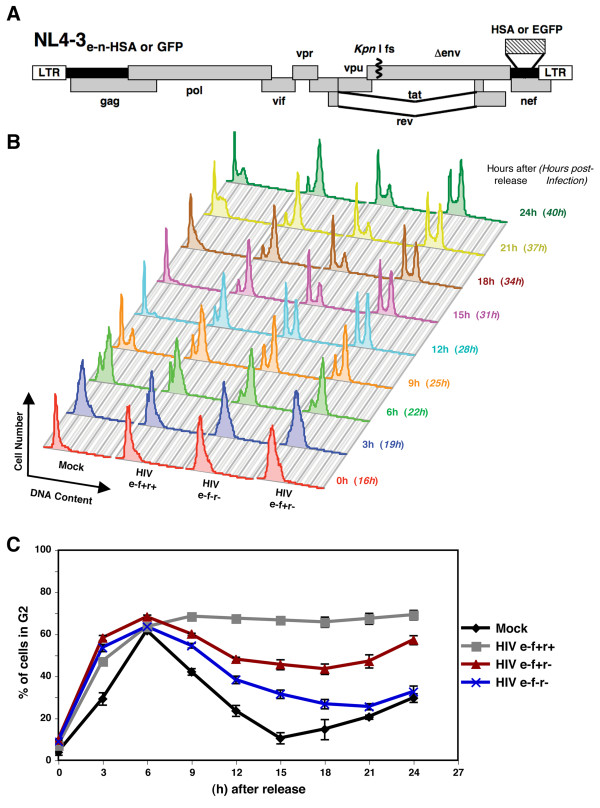
**Vif causes prominent G2 arrest in the absence of Vpr**. **(A) **Schematic of the NL4-3 HIV-1 molecular clones used. The NL4-3_e-n-HSA _(e-f+r+) lacks a functional *env *gene, due to a frameshift mutation, and the *nef *gene was replaced with HSA [3]. The NL4-3_e-n-GFP _has the same *env *frameshift, but the *nef *gene was replaced with EGFP [4,6]. The e-f+r- and e-f-r- mutants of NL4-3_e-n-HAS _and NL4-3_e-n-GFP _have been previously described [3,4]. **(B) **Jurkat cells were synchronized with a G1/S-phase blocker, aphidicolin, for 16 hours and then released for 10 hours prior to infection. The cells were blocked again at the time of infection with the following HIV-1 NL4-3_e-n-HSA _strains at an MOI of 5: e-f+r+, e-f+r-, or e-f-r-. DNA content was examined by flow cytometry using the cell permeable dye DRAQ5 (Biostatus) every 3 hours after release from the second aphidicolin blockade as previously described [3]. Infected cells highly expressing HSA and mock-infected cells are shown. These data are representative of three experiments using either the HSA- or GFP-expressing viruses. **(C) **The percentage of cells in the G2 phase of the cell cycle was graphed over the course of the experiment represented in panel B. Data are represented as the mean ± the standard deviation (SD) of quadruplicates and are representative of three experiments.

To investigate Vif-induced cell cycle arrest, we synchronized a Jurkat T cell line with the G1/S phase inhibitor, aphidicolin, and examined the DNA content of mock- and HIV-1-infected cells by flow cytometry [3]. Provirus expression was measured by the insertion of murine CD24 (heat stable antigen, HSA) or the enhanced green fluorescent protein (EGFP) into the Nef coding region (Figure 1A) [3,4,7]. Synchronized cells were released from aphidicolin after 16 hours of infection, and DNA content was monitored every 3 hours for 24 hours (Figure 1B and 1C). Cells infected with HIV-1_HSA _e-f+r+ (Env-negative, Vif-positive, Vpr-positive), expressing both Vif and Vpr proteins, progressed to the G2/M phase around 6 hours after release, similar to mock-infected cells (Figure 1B and 1C). Although mock-infected cells underwent mitosis and returned to G1 phase at 9 hours post-release, the majority of Vif+Vpr+ cells remained in G2/M phase for the duration of the experiment (24 hours) (Figure 1B and 1C). By comparison, the G2 arrest triggered by a e-f+r- virus was less dramatic than the e-f+r+ virus. Nevertheless, the infected cells showed striking G2 peaks that were sustained throughout the course of infection. Of note, the e-f-r- virus moderately delayed the cell cycle progression of infected cells, but failed to prevent cells from traversing back to G1 phase around 15-18 hours after the release (Figure 1B). These data demonstrate that Vif on its own was able to arrest cells at the G2 phase, but was less potent than cell cycle blockade by Vif and Vpr together.

**Figure 2:**
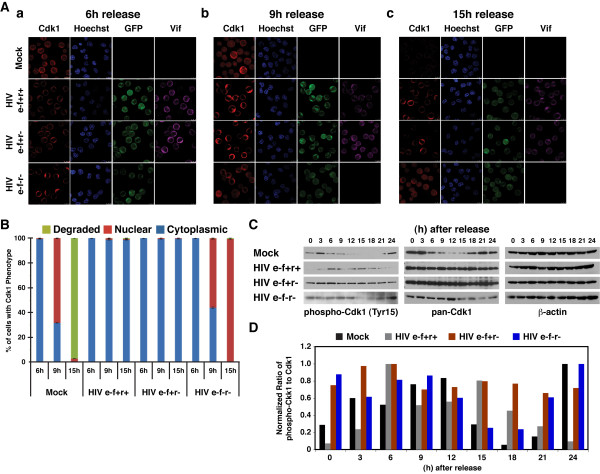
**Vif-induced dysfunction of Cdk1**. Jurkat cells were synchronized and infected as in Figure 1 with the GFP-expressing viruses. These data are representative of three experiments with infection efficiencies ranging from 85-95% based on GFP expression. **(A) **Nuclear translocation of Cdk1 was barely detectable in Vif-expressing cells. Vif and Cdk1 localization patterns were visualized by immunofluorescent confocal microscopy using the following antibodies: rabbit anti-Vif (AIDS Research and Reference Reagent Program [ARRRP]) [53], mouse anti-Cdk1 (anti-cdc2, Santa Cruz Biotechnology), goat anti-rabbit-Alexa565 (Molecular Probes), and goat anti-mouse-Alexa647 (Molecular Probes). GFP, expressed by infected cells, was measured by direct fluorescence. Nuclei were counterstained with Hoechst 33342 (Molecular Probes). **(B) **At least 350 cells were counted from representative fields, and the percentage of cells showing the indicated phenotypes for Cdk1 were plotted at each time point. **(C) **Cdk1 is phosphorylated (inactivated) in Vif-expressing cells. Bulk lysates were prepared at the indicated time points and analyzed for inhibitory Cdk1 Tyr15 phosphorylation by immunoblotting using a rabbit anti-phospho-Cdk1 Tyr15 antibody (anti-phospho-cdc2 Tyr15, Cell Signaling Technology). Total Cdk1 expression was examined using a mouse anti-Cdk1 antibody (anti-cdc2, Santa Cruz Biotechnology) on the same blot after stripping off the phospho-Cdk1 antibody. An immunoblot using a mouse-anti β-actin antibody (anti-β-actin, Sigma-Aldrich) is provided as a loading control. **(D) **Densitometry of the bands in panel C was performed using ImageJ (NIH), and the intensity of each band was normalized to β-actin. The normalized ratio of phospho-Cdk1 to total Cdk1 was plotted for each time point.

To elucidate the molecular defects causing cell cycle arrest in Vif-expressing cells, we examined the translocation of MPF, which occurs at the G2/M phase transition. Synchronized Jurkat cells were examined at 3-hour intervals post-release for the subcellular localization of Cdk1 by confocal immunofluorescence microscopy as previously described (Figure 2A and 2B)[39]. In mock-infected cells, Cdk1 was mostly cytoplasmic at 6 hours post-release. It then translocated into the nucleus at 9 hours, prior to disappearing presumably due to proteasomal degradation (Figure 2A and 2B). By contrast, Cdk1 remained essentially cytoplasmic in cells infected with either the e-f+r+ or the e-f+r- virus (Figure 2Aa-c and 2B). The cells infected with the e-f-r- virus, lacking both Vif and Vpr, exhibited similar nuclear translocation of Cdk1 as the mock-infected cells, but with delayed kinetics (Figure 2Aa-c and 2B). Thus, our data suggest that Vif inhibits Cdk1 nuclear translocation whether or not Vpr is present.

Activation of Cdk1 is regulated by phosphorylation [18,21]. Inactive Cdk1 remains cytoplasmic with inhibitory phosphates attached to Thr14 and Tyr15 until cells clear the G2 checkpoint [27,40-42]. Because of the persistent cytoplasmic localization in HIV-infected cells, we examined the phosphorylation status at Tyr15 of Cdk1 by western blot analysis as previously described [6]. In mock-infected cells, Tyr15 phosphorylation increased at 3 hours post-release, coinciding with the S to G2 phase transition (Figure 1B and 2B). Then, after 6 hours, the phosphorylated form, as well as the total amount of Cdk1, started to decline (Figure 2C and 2D). However, the ratio of phosphorylated to total Cdk1 continued to increase until 12 hours post-release (Figure 2D). This ratio then decreased until 18 hours post-release when both the phosphorylated form and the total amount of Cdk1 were barely detectable (Figure 2C and 2D). These data suggest that Cdk1 had become active, carried out its mitosis promoting function, and undergone degradation. Cells infected with the e-f+r+ virus maintained constant Cdk1 protein levels but showed inconsistent Tyr15 phosphorylation (Figure 2C and 2D). However, Cdk1 was strongly phosphorylated and remained undegraded throughout the course of the experiment in cells infected with the e-f+r- virus (Figure 2C and 2D). Interestingly, Tyr15 phosphorylation was more pronounced in Vif-induced G2 blockade (in the absence of Vpr). While previous studies have shown that cells expressing Vpr have more phosphorylated Cdk1 than normal cells [43-45], Vpr can also increase phosphorylation of Cdk1 at Thr14 as well as Tyr15 [43]. This may explain the discrepancy in the Tyr15 status between cells infected with the e-f+r+ virus versus the e-f+r- virus in our experiments. Perhaps the Cdk1 in the cells infected with the e-f+r+ virus, expressing both Vpr and Vif, is still inactive due to Thr14 phosphorylation. The phosphorylation of Cdk1 Tyr15 in cells infected with the e-f-r- virus was similar to the mock-infected cells, with increased and decreased phosphorylation following the stages of the cell cycle (Figures 1B and 2C and 2D). These data suggest that Vif can directly impede Cdk1 activation and subsequent nuclear translocation.

**Figure 3:**
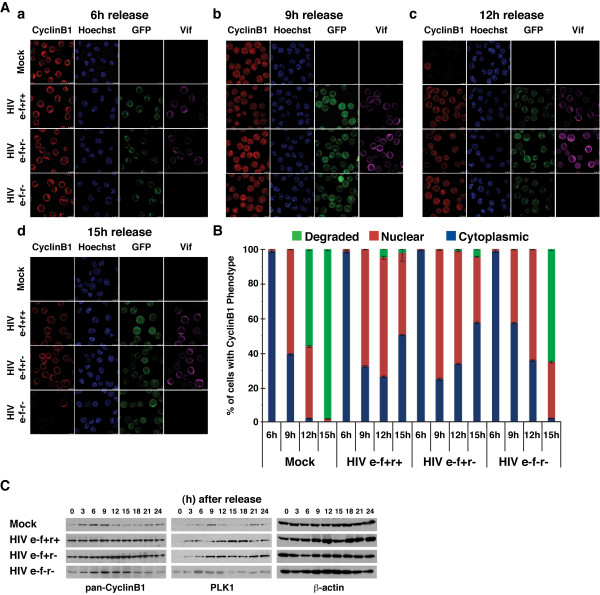
**Vif-induced abnormalities in CyclinB1 and PLK1**. Jurkat cells were synchronized and infected as in Figure 1 with the GFP-expressing viruses. These data are representative of three experiments with infection efficiencies ranging from 85-95% based on GFP expression. **(A) **CyclinB1 localizes to the nucleus in Vif-expressing cells, but is not degraded normally. Subcellular localization of CyclinB1 and Vif was determined by immunofluorescent confocal microscopy as in Figure 2 panel A using a mouse anti-Vif antibody (ARRRP) [54-56] and a rabbit anti-CyclinB1 antibody (Santa Cruz Biotechnology). **(B) **At least 350 cells were counted from representative fields, and the percentage of cells showing either a degraded, nuclear, or cytoplasmic phenotype for CyclinB1 were plotted at each time point. **(C) **CyclinB1 degradation is not observed in infected cells, and PLK1 expression is elevated in infected cells. A duplicate blot from Figure 2 panel C was probed with a mouse anti-CyclinB1 antibody (Cell Signaling Technology). PLK1 expression was examined using a mouse anti-PLK1 antibody (Upstate/Millipore). The expression of β-actin using a mouse-anti β-actin antibody (anti-β-actin, Sigma-Aldrich) is provided as a loading control.

We also investigated the effect of HIV-1-induced cell cycle arrest on CyclinB1. Immunofluorescent staining of mock-infected cells showed that CyclinB1 translocates from the cytoplasm (6 hours post-release) to the nucleus (9 hours) (Figure 3Aa-b and 3B). Similar to Cdk1, CyclinB1 was evidently degraded and almost undetectable after 12-15 hours when cells re-entered the G1 phase (Figures 1B and 3Ac-d and 3B). This was expected since CyclinB1 is known to be degraded upon exit from mitosis [46-52]. Intriguingly, unlike Cdk1, CyclinB1 retained the ability to translocate into the nucleus by 9 hours in cells infected with either the e-f+r+ or the e-f+r- virus (Figure 3Ab and 3B). In addition, CyclinB1 levels persisted in infected cells throughout the course of the experiment (Figure 3A and 3B). However, after 12 hours, many HIV-infected cells that expressed Vif showed CyclinB1 had returned to the cytoplasm (Figure 3Ac-d and 3B). Cells infected with the e-f-r- virus, which do not express Vif or Vpr, exhibited similar CyclinB1 translocation and degradation as mock-infected cells, but with delayed kinetics (Figure 3A and 3B). Western blot analysis confirmed the findings from microscopy. The levels of CyclinB1 in mock-infected cells increased when cells were in G2 phase (6 hours), declined when cells were in G1 and S phases (12-18 hours), and began to increase again after 21 hours (Figures 1B and 3C). By contrast, CyclinB1 levels remained stable throughout the entire time course in HIV-1-infected cells that expressed Vif (Figure 3C). Similar to the confocal data, the levels of CyclinB1 in cells infected with the e-f-r- virus followed a similar, but delayed pattern compared to mock-infected cells (Figure 3C).

Because CyclinB1 retained the capacity to enter the nucleus in arrested cells expressing Vif, in the presence or absence of Vpr, we also examined PLK1, which phosphorylates the CyclinB1 CRS to target it to the nucleus [35]. In mock-infected cells and cells infected with the e-f-r- virus, PLK1 expression peaked around 6-9 hours, occurring before and during the nuclear accumulation of CyclinB1 as its published role would suggest. PLK1 expression then decreased at 12-18 hours, when cells progressed through mitosis (Figure 1B and 3C). Cells infected with either Vif-expressing virus exhibited an abnormal phenotype. Once PLK1 expression was induced between 3 and 12 hours, the levels remained elevated, possibly due to the G2 cell cycle arrest (Figure 3C). This increased expression of PLK1 could possibly explain the ability of CyclinB1 to still translocate into the nucleus. However, PLK1 expression remained elevated when CyclinB1 returned to the cytoplasm after 12 hours (Figure 3). This may be due to a difference in binding to the 14-3-3θ scaffold protein (increased for CyclinB1 and decreased for PLK1) as we have previously shown [6].

**Figure 4:**
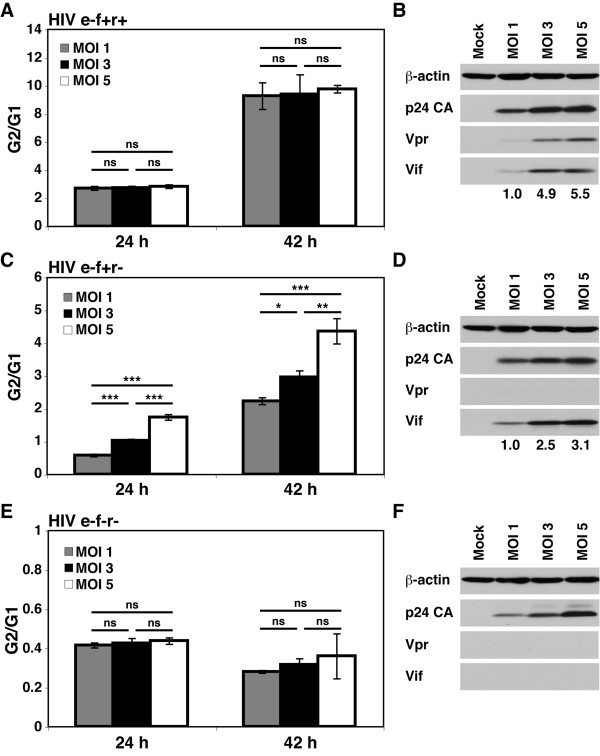
**Vif-induced cell cycle arrest is partially dependent on MOI**. Non-synchronized Jurkat cells were infected with NL4-3_e-n-GFP _e-f+r+ (A and B), e-f+r- (C and D), and e-f-r- (E and F) at the indicated MOIs. **(A, C, and E) **DNA content of GFP^+ ^cells was examined by flow cytometry using DRAQ5 at 24 and 42 hours post-infection as previously described [4]. The percentage of the G2 and G1 populations were modeled using the Watson Pragmatic cell cycle model and the ratio was plotted [4]. All data were represented as mean ± the SD of triplicates. The ns, single (*), double (**), and triple (***) asterisks denote p > 0.05, p < 0.05, p < 0.01, and p < 0.001, respectively, using a one-way analysis of variance (ANOVA) with multiple-comparison tests (Prism, Graph-Pad Software). For each MOI at each time point the G2/G1 ratio for e-f+r+>e-f+r->e-f-r- with p < 0.00001 as analyzed by a one-way ANOVA with multiple-comparison tests. **(B, D, and F) **The expression of Vif and Vpr increases with increasing MOIs. Lysates were prepared from infected cells at 24 hours post-infection and analyzed for the expression of viral proteins by immunoblotting. The following antibodies were used: mouse anti-p24-capsid (ARRRP) [55,57], rabbit anti-Vpr (a kind gift from B. Sun), mouse anti-Vif (ARRRP) [54-56], and mouse-anti-β-actin (Sigma-Aldrich). Densitometry of the bands was performed using ImageJ (NIH), and the intensity of each band was normalized to β-actin. The fold change of Vif expression is shown under the immunoblots. These data are representative of three experiments.

A recent study reported that Vif induced a delay in cell cycle rather than complete arrest [15]. It is difficult to compare our results and those reported by DeHart and colleagues because the experimental system used in their study, especially the cells (SupT1 cells) and level of infection (multiplicities of infection [MOI] of 1-2), is different from ours [3,15]. We observed that the G2 arrest due to Vif alone was most pronounced at a high MOI. Furthermore, unlike the combination of Vpr and Vif, Vif-induced cell cycle arrest showed a direct relationship with increasing MOI (and therefore the increasing expression level of Vif), whereas the arrest caused by the e-f+r+ virus appeared to be independent of MOI (Figure 4A-D). However, similar to the cell cycle blockade caused by the e-f+r+ virus, cells infected with the e-f+r- virus showed the highest G2/G1 ratio on day 2 post-infection, when the expression of the provirus peaks (Figure 4 and data not shown). We observed strong cell cycle arrest caused by high levels of Vif expression in both synchronized and non-synchronized Jurkat cells (Figures 1B and 4B). As previously shown [3], the e-f-r- virus caused no significant G2 arrest (Figure 4E and 4F). Thus, high expression of Vif arrested cells in the G2 phase, although not to the same degree as the combined expression of Vpr and Vif.

The HIV-1 accessory proteins Vif and Vpr block cells at the G2 phase of the cell cycle [3]. We now provide some molecular insights on how Vif induces cell cycle arrest. Our study strongly suggests that Cdk1-CyclinB1 dysregulation accounts for Vif-mediated G2 blockade. However, the precise mechanism of this dysfunction remains to be determined. Intriguingly, cells infected with the e-f+r+ or the e-f+r- viruses showed differences in phenotypes, especially the status of Cdk1, likely indicating different mechanisms of action for the two proteins.

Why HIV-1 has evolved two molecularly different mechanisms for G2 inhibition is an important unanswered question. Both forms of arrest could be byproducts of viral metabolism. Alternatively, it may be that the G2 phase is so important for a productive viral infection cycle that the virus must ensure G2 cell cycle arrest by two distinct mechanisms. In either case, both Vif and Vpr are major players in HIV-1 cytopathicity, and virus-induced cell cycle inhibition may be intrinsically related to viral pathogenesis. Consistent with this possibility, our previous work showed that both Vif and Vpr can independently contribute to HIV-1 cytopathicity [3]. It will be important to determine how the specific molecular pathways converge in necrotic death of arrested, infected T-cells.

## List of Abbreviations

HIV-1: human immunodeficiency virus type 1; HSA: heat-stable antigen; MOI: multiplicity of infection; PLK1: polo-like kinase 1; Thr: threonine; Tyr: tyrosine; Vif: viral infectivity factor; Vpr: viral protein R.

## Competing interests

The authors declare that they have no competing interests.

## Authors' contributions

KS, RAB, and MJL designed the study. KS, RAB, BCD, and NB carried out the experiments. KS, RAB, BCD, NB, and MJL interpreted data. KS, RAB, and MJL wrote the manuscript. MJL provided financial support. All authors read and approved the final manuscript.
